# Biodegradability and platelets adhesion assessment of magnesium-based alloys using a microfluidic system

**DOI:** 10.1371/journal.pone.0182914

**Published:** 2017-08-10

**Authors:** Lumei Liu, Youngmi Koo, Boyce Collins, Zhigang Xu, Jagannathan Sankar, Yeoheung Yun

**Affiliations:** 1 National Science Foundation-Engineering Research Center for Revolutionizing Metallic Biomaterials, North Carolina Agricultural and Technical State University, Greensboro, North Carolina, United States of America; 2 FIT BEST Laboratory, Department of Chemical, Biological, and Bioengineering, North Carolina Agricultural and Technical State University, Greensboro, North Carolina, United States of America; University of North Texas, UNITED STATES

## Abstract

Magnesium (Mg)-based stents are extensively explored to alleviate atherosclerosis due to their biodegradability and relative hemocompatibility. To ensure the quality, safety and cost-efficacy of bioresorbable scaffolds and full utilization of the material tunability afforded by alloying, it is critical to access degradability and thrombosis potential of Mg-based alloys using improved *in vitro* models that mimic as closely as possible the *in vivo* microenvironment. In this study, we investigated biodegradation and initial thrombogenic behavior of Mg-based alloys at the interface between Mg alloys’ surface and simulated physiological environment using a microfluidic system. The degradation properties of Mg-based alloys WE43, AZ31, ZWEK-L, and ZWEK-C were evaluated in complete culture medium and their thrombosis potentials in platelet rich plasma, respectively. The results show that 1) physiological shear stress increased the corrosion rate and decreased platelets adhesion rate as compared to static immersion; 2) secondary phases and impurities in material composition induced galvanic corrosion, resulting in higher corrosion resistance and platelet adhesion rate; 3) Mg-based alloys with higher corrosion rate showed higher platelets adhesion rate. We conclude that a microfluidic-based *in vitro* system allows evaluation of biodegradation behaviors and platelets responses of Mg-based alloys under specific shear stress, and degradability is related to platelets adhesion.

## Introduction

Biodegradable cardiovascular stents provide initial mechanical support to diseased arteries, such as atherosclerosis. Magnesium (Mg)-based scaffolds are being investigated to enlarge the position of atherosclerosis because of their biodegradability and biocompatibility [1, 2]. Mg-based alloys can degrade completely in vascular and avoid second surgical intervention to remove the implant. Mg-based scaffolds such as bioabsorbable metal scaffold (AMS-1) and drug-eluting magnesium-alloy scaffold (DREAMS) [3], have been tested in clinical trials in patients with coronary artery disease. The trials thus far demonstrate the treatments are able to avoid long-term clinical problems occurring in permanent stents, such as further thrombosis, permanent physical irritation, long-term endothelial dysfunction, and inability to adapt to growth [4–7]. As the number of successful clinical applications of biodegradable metals grows, an opportunity exists to tailor device properties through designed choice of alloying elements and sample preparation. Ideally, we can screen these alloys at the material development stage to reduce costs and increase the likelihood of clinical success.

However, a challenge to the widespread application of Mg alloys to biomedical applications remains. The high electrochemical activity of Mg makes the corrosion behavior unpredictable *in vivo* influenced by complex environmental factors (e.g. temperature, dynamic flow and surrounding ion composition). *In vivo* and *in vitro* test results of Mg and Mg alloy corrosion typically show differences and even opposite outcomes [8]. For example, the average corrosion rate of pure Mg (>99 wt.%) varies from 0.15 to 1.68 mm/year with different immersion solutions [8]. Pure Mg (>99 wt.%), LAE442 and AZ91D showed different corrosion rates achieved from corrosion tests *in vitro* (ASTM standard immersion) and *in vivo*, [9, 10]. Current ASTM standard *in vitro* corrosion tests (e.g. ASTM-G31-72 [11] and ISO 10993 series [12]) are not appropriate to predict corrosion behavior of Mg alloys *in vivo*.

There is an increasing demand for reliable and controllable *in vitro* model(s) to simulate *in vivo* degradation environment and to evaluate biodegradability and thrombosis potential of Mg-based alloys influenced by both element composition and environmental factors. The microstructure and micro-constituent features of Mg-based alloys, as innate factors, affect degradation behavior. For instance, the presence of impurities and secondary phases acting as local cathodes cause local galvanic couples and accelerate corrosion [13]. Mg-based alloys are highly susceptible to micro-galvanic corrosion, because impurities like nickel (Ni), iron (Fe) and copper (Cu) form efficient cathodes for galvanic corrosion of magnesium [14–16]. Both pitting corrosion and filiform corrosion are observed in Mg-based alloys containing binary phases [17–20]. The degradation rate is subject to environmental factors such as flow-induced shear stress and hemodynamics and varies in different implantation positions in cardiovascular system [21–23]. It was demonstrated that the fluid flow has a significant impact on the degradation of absorbable metallic stents, including degradation kinetics, degradation modes, degradation rates, degradation products, and local pH changes [24, 25]

Besides biodegradability, Mg-based alloys have been proved hemocompatible with low thrombogenicity [26]. WE43 stent has been demonstrated safe *in vivo* and did not show particle embolization, thrombosis, excess inflammation, or fibrin deposition [27]. An ideal biocompatible stent should exhibit no platelet adhesion, be non-thrombogenic, anti-inflammatory and promote healing. These properties vary for different Mg-based alloys and are typically probed by measuring platelet adhesion rates [28, 29]. Platelets adhesion is one of the most important factors to evaluate thrombosis potential of cardiovascular stents, because platelet adhesion is the trigger for blood coagulation causing thrombus formation [30] [31]. There are two primary potential causes for thrombosis when utilizing Mg-based alloys. Mg-based alloys with poor corrosion behavior and hemocompatibility may recruit aggregated platelets resulting in thrombus growth and nidus restenosis [32]. Secondly, physiologically emboli (particulate-related) or undissolved residues can be produced during degradation process and cause downstream thrombosis [33].

In terms of biodegradability and initial thrombogenic potential, a realistic *in vitro* environment is a prerequisite to simulate *in vivo* hemodynamic microenvironment. In this study, a novel test bed was developed to evaluate the safety and quality screening of Mg-based alloys for cardiovascular stents applications. This study utilizes a microfluidic platform which mimics a small blood vessel to evaluate biocompatible/biodegradable properties of Mg-based alloys [34], specifically to investigate the interface between Mg-based alloys and a simulated physiological environment: dynamic cell culture medium and platelet rich plasma. Platelets adhesion tests have been conducted in static condition [28, 29], but not fully studied in dynamic condition. This microfluidic test bed was designed to evaluate Mg-based alloys in simulated stent application conditions for a better estimate of *in vivo* degradation behavior and thrombosis of Mg-based scaffolds.

## Material and methods

### Alloys preparation

Magnesium-based alloys WE43, AZ31, ZWEK-L and ZWEK-C and stainless steel 316L SS (composition shown in Table 1) were chosen to evaluate the degradation behavior in a blood vessel simulated microfluidic system. Stainless steel (316L SS) was purchased from McMaster-Carr (Douglasville, GA). WE43 Mg casting alloy was purchased from Magnesium Elektron North America Inc. (Manchester, NJ). Extruded AZ31 Mg alloy was purchased from Goodfellow (Oakdale, PA). ZWEK was fabricated with 99.97% Mg, 99.99% Zn and master alloys Mg-30%Y, Mg-30% Rare earth (Dy) and Mg-30%Zr in NSF-funded ERC for Revolutionizing Metallic Biomaterials (RMB) at NC A&T State University. The ZWEK alloy fabrication procedure (io: http://dx.doi.org/10.17504/protocols.io.iepcbdn.io.[PROTOCOL DOI]) was similar to that reported previously [35]. ZWEK-L and -C samples were cut from the longitudinal and cross-sectional directions from the extruded rod, respectively. These alloys were cut and polished into cuboids, 5 mm in length, 2 mm in width and 3 mm in thickness. The samples were mechanically polished with silicon carbide (SiC) paper progressively up to 1000 grit with water and then polished with 1200 grit SiC paper with isopropyl alcohol. Specimens were then ultrasonically cleaned sequentially with acetone and ethanol, and dried using compressed air.

**Table 1:** Chemical compositions of tested alloys (wt.%).

316L SS		WE43		AZ31		ZWEK
Chromium, Cr	16–18.5%	Yttrium, Y	4%	Aluminium, Al	3.533%	Magnesium, Mg
Nickel, Ni	10–15%	Rare Earths	3%	Zinc, Zn	1.167%	Zinc, Zn
Carbon, C	0–0.08%	Zirconium, Zr	0.5%	Manganese, Mn	0.242%	Dysprosium, Dy
Manganese, Mn	0–2%	Magnesium, Mg	Balance	Iron, Fe	0.007%	Yttrium, Y
Copper, Cu	0–1%			Silicon, Si	0.0008%	Zirconium, Zr
Molybdenum, Mo	0–3%			Nickel, Ni	0.002%	
Silicon, Si	0–1%			Magnesium, Mg	Balance	
Sulfur, S	0.35%					
Phosphorus, P	0–0.045%					
Nitrogen, N	0–0.1%					
Titanium, Ti	0.7% Max.					
Iron, Fe	58.23–73.61%					

The composition of 316L SS and WE43 are provided by the companies. The composition of AZ31 was analyzed by X-ray fluorescence (XRF). And ZWEK composition is not shown due to confidentiality.

### Microstructure observation and measurement

The alloy grain structure was observed under a field emission scanning electron microscope (SEM, SU8000, Hitachi, Japan) after etching with 20% natal and acetic picral solution (10mL acetic acid + 4.2g picric acid + 10 mL distilled water + 70 mL 95% ethanol). Grain sizes were measured using ImageJ software (US National Institutes of Health, Bethesda, MD). Impurities were detected by electron dispersive X-ray spectroscopy (EDX, Bruker AXS5350, Germany).

### Microfluidic system design and fabrication

**Fig 1:**
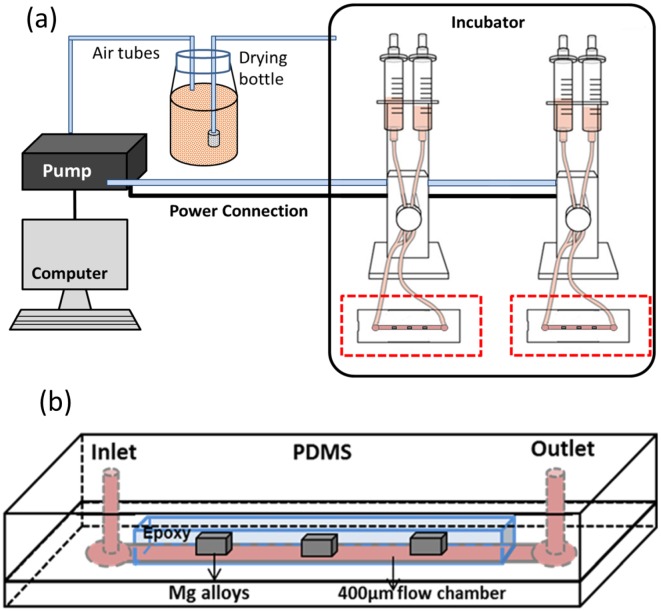
Microfluidic system design. (a) A schematic setup of the microfluidic system (b) A diagram of the plate flow chamber with reusable epoxy-embedded alloys.

Fig 1(a) shows a pump system (ibidi^®^, München, Germany) for simulation of blood flow microenvironment. The system is able to provide a range of 4 ~ 37.1 dyne/cm^2^ shear stress for standard 0.4 μ-slide flow chamber (50 x 5 x 0.4 mm^3^ ibidi^®^, München, Germany). As shown Fig 1(b), microfluidic chip was designed to simulate blood vessel which Mg alloys implanted. The fabrication of microfluidic chip mold followed the protocol of soft-lithography negative photoresist process for SU-8 2100 (Microchem Corp., USA) [36]. Briefly, the SU-8 photoresist was spin-coated on Si (1000 rpm, 30 s, with the acceleration of 300 rpm/second) and soft-baked on a hot plate (65°C, 10 min, followed by 95°C, 120 min). The SU-8 coated Si was exposed through a photomask with a Solitec mask aligner (50mW/cm^2^, 18 s). Post exposure bake was done on a hotplate (65°C, 5 min, followed by 95°C, 30 min). The final sample was immersed into SU-8 developer for 15 min. The final master was cleaned with isopropyl alcohol and stored at room temperature for future use.

To form the polymeric chambers, polydimethylsiloxane (PDMS; Dow Corning, Midland, MI, USA) elastomer was mixed (1:10 w/w ratio), poured onto the negative master wafer, degassed, and allowed to cure overnight. The thickness of the chambers was 400μm as confirmed by micro X-ray computed tomography (CT, GE Phoenix Nanotom-M^™^, GE Sensing & Inspection Technologies GmbH). Three alloy cuboids (about 5 x 3 x 2 mm^3^) were also mounted into epoxy resin (Epokwick^®^ Epoxy resin, Buehler, USA) distributed at 1 cm intervals. The Epoxy was cut into a long brick (about 45 x 5 x 5 mm^3^) and embedded into PDMS in a petri dish. PDMS replicas were then pulled off the wafer and petri dish. The two parts of the PDMS were attached together after polishing the surface of the implants. The epoxy embedded 3-alloy with freshly polished surface was used for multi-screening under the same environment. The inlet and outlet holes were punched with a 19-gauge blunt-nose needle. After connection with ibidi pump system, the samples were tested 3 days in an incubator with 37°C, 5% CO_2_. Each alloy was tested at least three times.

Additionally, computational fluid dynamics (CFD) simulation was used to predict velocity distribution on the Magnesium alloys and 316L SS surface and pressure contour in the chamber using COMSOL Multiphysics^®^ (S1 Fig). Dulbecco's modified eagle medium (DMEM) with supplements (10% fetal bovine serum and 1% penicillin/streptomycin) and platelets rich plasma (PRP) were used as running medium. Flow rate (Q) was set at 6.63 ml min^-1^ for DMEM and 4.70 ml/min for PRP to simulate the experimental conditions, yielding a shear stress 6.81 dyne/cm^2^ to mimic *in vivo* coronary artery mean wall shear stress [21]. For static condition, the other set of samples was immersed in complete DMEM for 3 days and PRP for 3 hours.

### Corrosion characterization

To characterize corrosion behavior, micro X-ray computed tomography was conducted on samples before and after testing in microfluidic system and after removing corrosion product on alloys surface by chromic acid dip. The X-ray emission parameters of the CT included a voltage of 120 kV and current of 80 μA. The two-dimensional (2D) planes and the three-dimensional (3D) models were reconstructed using Phoenix datos|x software. The volume ratio of residual sample to the initial samples, and the volume ratio of corrosion products to residual sample were calculated from CT data analysis using VG Studio Max software (v 2.1). Corrosion rates (mm/y) are provided, but it should be noted that localized corrosion was not considered and therefore the calculated corrosion rates are rough estimates. Corrosion rates based on the reduction of the metallic volume after corrosion were calculated from the obtained 3D data. Assuming uniform and surface corrosion mechanisms, the reduction of the implant volume could be converted into a corrosion rate by using the following of equation [9]:

$$\text{CR}=\frac{\Delta V}{At}$$

where CR represents corrosion rate, ΔV is the volume loss which is equal to the difference between initial and residual volume, A is the implant surface area and t is the exposure time.

After 3 days’ test in complete DMEM, samples were used to analyze the chemical compositions of the corrosion products using a field emission scanning electron microscope (SEM, SU8000, Hitachi, Japan) and electron dispersive x-ray spectroscopy (EDX, Bruker AXS5350, Germany) after sputter coating. The circulated DMEM was collected and pH was measured. Then the collected medium was centrifuged at 5000g, 25°C for 20 minutes. After pipetting out the supernatant, the sediment was put in a freeze dryer (Freezone, Labconco, USA) for 24 hours. The freeze-dried sediment was analyzed by EDX.

### Platelet adhesion test

Platelet adhesion experiment was carried out to evaluate the initial stage of surface thrombogenicity of the samples and to examine the interaction between platelets and the materials *in vitro* [37]. The area of all of the samples applied to *in vitro* experiments was approximately 2 mm x 5 mm. Platelet-rich-plasma (PRP) is human fresh normal peripheral blood platelets. The donor is male, 27 years old, and negative in human immunodeficiency virus (HIV), hepatitis B virus (HBV), hepatitis C virus (HCV). The product was purchased from Allcells^®^ with the concentration of 5 x 10^8^/ml. PRP was used immediately upon arrival. Micro-Renathane tubing (0.066-in. ID, 0.095-in. OD; Braintree Scientific, Braintree, MA) was used as platelet-rich plasma tubing, because it is a polyurethane-based tubing and polyurethane is flexible and highly blood compatible, thus minimizing clotting [38–40]. The samples were exposed to PRP for 3 hours.

Following perfusion and static immersion, samples were gently rinsed with PBS 3 times to remove non-adhesive platelets and incubated with glutaraldehyde (2.5% in PBS; Sigma–Aldrich) for 10 min. Next, the materials were washed three times with PBS and dehydrated in consecutive stages of increasing ethanol concentrations. The samples were then chemically dried with hexamethyldisilazane (Thermo Fisher). Finally, the samples were coated with gold-palladium and scanning electron micrographs (SEMs) were obtained.

### Statistical analysis

Statistical analysis (t-test) of corrosion rate (mm/year) and platelet adhesion rate (platelets/mm^2^) was performed using Microsoft Excel. The correlation between corrosion rate and platelet adhesion rate was plotted with GraphPad Prism^®^ Version 5.0. Significance was established at p < 0.05. Data are expressed as mean ± standard deviation (SD).

## Results and discussion

### Corrosion behavior evaluation

#### Corrosion rate

**Fig 2:**
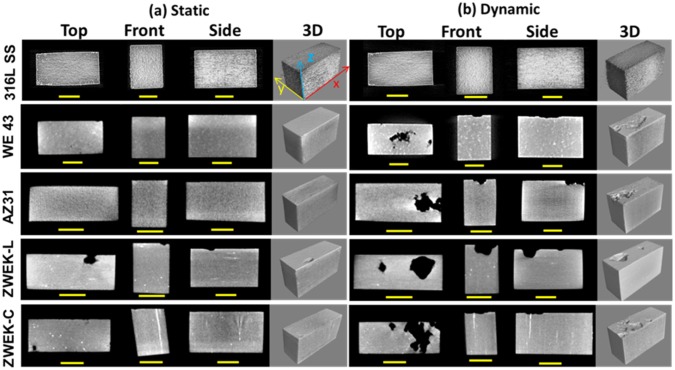
Micro-CT images of selected cross-sections morphology and 3D view. The cross- section view of the top is XY plane, the front is YZ plane, the side is XZ plane. The degraded alloys were tested at both static (a) and dynamic (b) conditions after removing corrosion product by chromic acid. Scale bar = 1.5mm.

**Fig 3:**
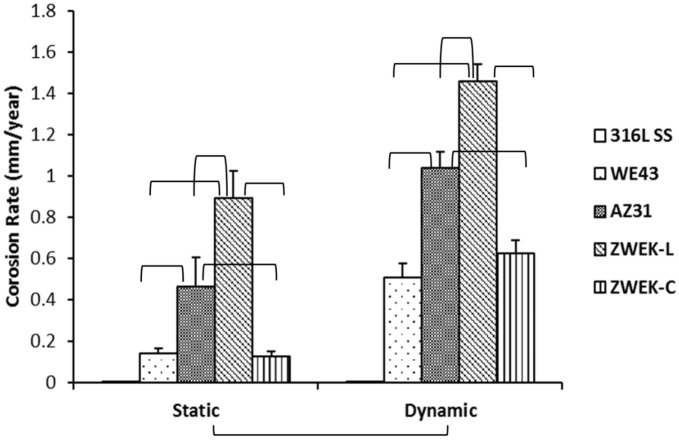
Statistical analysis of corrosion rate of four Mg-based alloys at both static and dynamic conditions. Parenthesis mean significant differences between groups (P<0.05).

Corrosion morphologies were collected from X-ray CT data of the samples after removing corrosion product on the 3rd day (Fig 2). The corrosion morphologies provide a 2D cross-section from the view of the top (XY plane), front (YZ plane) and side (XZ plane) and a 3D view of the corroded alloys blocks. The selected cross-section of XY plane is at the most corroded surface and the YZ and XZ plane views are at the deepest corroded site. This assignment was determined by comparing the scanned images before and after corrosion product removal by cleaning in chromic acid. The degradation rates calculated based on the CT data according to equation (1) are presented in Table 2 and Fig 3. The corrosion morphologies and rates shows that 1) the alloy’s surface experienced more corrosion at dynamic condition (shear stress = 6.81 dyne/cm^2^) than static condition (shear stress = 0); 2) at both static and dynamic condition, ZWEK-L had the most severe corrosion and WE43 had the least corrosion; 3) from static to dynamic condition, corrosion rate of WE43 and ZWEK-C was increased more magnitude than AZ31 and ZWEK-L. This indicates that dynamic affected WE43 and ZWEK-C more magnitude than AZ31 and ZWEK-L. The higher corrosion rate under dynamic condition compared with static condition is attributed to fluid dynamics on the corrosion behavior including mass transfer and shear stress. The flow accelerates the arrival of the corrosive medium with aggressive Cl^-^ to the Mg surface and removes the corrosion products [41], and accelerates the transfer of Mg^2+^, OH^-^, and other ions that may form solid corrosion products from the metal/solution interface to the bulk solution, aggravating the corrosion. The metal surface refreshed by dynamic culture medium promotes degradation [42]. Additionally, mass transfer induces the pressure-driven diffusion caused by concentration gradients and is accelerated by high mechanical force from flow-induced shear stress [25].

**Table 2:** Corrosion rate of four Mg-based alloys and 316L stainless steel at both static and dynamic conditions in DMEM for 3 days.

Corrosion rate in DMEM (mm/year)	316L SS	WE43	AZ31	ZWEK-L	ZWEK-C
Static	0	0.14 ±0.02	0.47 ±0.14	0.90 ±0.13	0.13 ±0.02
Dynamic	0	0.51 ±0.07	1.04 ±0.08	1.46 ±0.08	0.63 ±0.06

**Fig 4:**
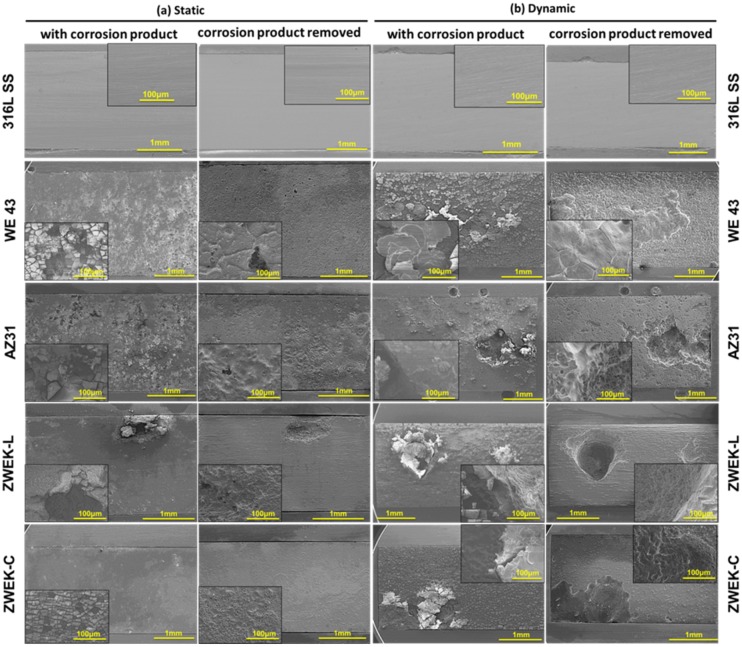
Surface images of corroded sample implants before and after removing corrosion products. The images display the surface morphologies at both static (a) and dynamic (b) conditions.

In 2D selected cross-sections of XY, YZ and XZ plane of micro-CT results (Fig 2), the size of the cavities fit the corrosion rate (WE43≈ZWEK-C<AZ31<ZWEK-L, P<0.05) visually. WE43 has the smallest cavities and ZWEK-L has the largest cavities. The pitted cavities are much deeper on ZWEK-L than ZWEK-C. This is confirmed by the surface morphologies of corroded alloys observed by SEM (Fig 4). Though ZWEK-C and ZWEK-L have the same elemental composition, the cross section surface of ZWEK exposed to flow corroded much slower than longitude surface. These observations support the assertion that composition is an important factor that affects the quality of biodegradable alloys and very important for their corrosion behavior [43–45], but also provides a consideration extruded materials application to scaffold material for stents. The corrosion resistance is increased when surfaces perpendicular to the extruded direction face the lumen of vascular, than the surface parallel to the extruded direction. The reason is related to impurities and secondary phases (Explained in section: Element composition-secondary phase and impurities).

#### Corrosion type and corrosion product

The corrosion morphologies of tested Mg-based alloys before and after removing corrosion products were observed under SEM (Fig 4). The surface morphologies of all tested samples display different degrees of pitting corrosion and localized corrosion. At static immersed condition, corrosion is usually localized [15]. Under dynamic condition, the induced-shear stress of the WE43, AZ31, ZWEK-L, and ZWEK-C made the sample surface more susceptive to pitting and localized corrosion (Figs 2 and 4). Pitting corrosion preferably occurs in chloride-ion-enriched environments such as in biological systems [46]. The dynamic flow, as the main biophysical environment, affects the corrosion behavior of magnesium-based alloys through applying shear stress to the surface and promoting diffusion and Cl^-^ replenishment.

**Fig 5:**
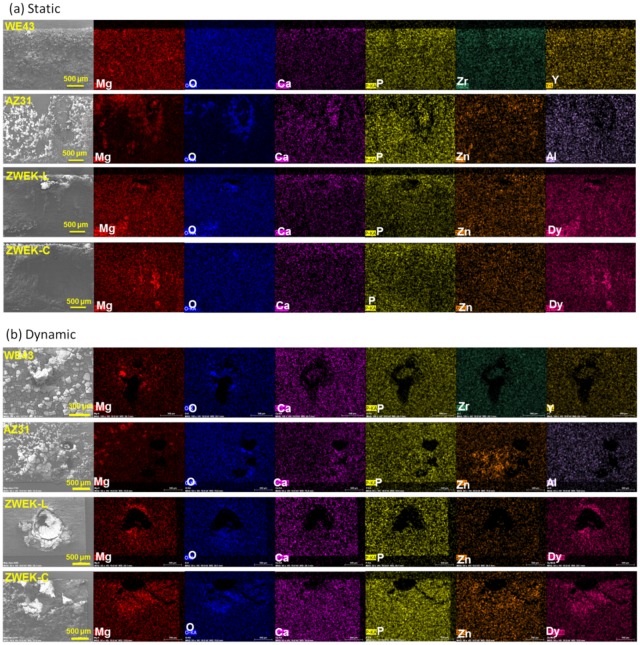
EDX images of elemental distribution. The elemental distribution was observed by EDX at the corrosion position of Mg-based alloys after 3 days test at static (a) and dynamic (b) conditions.

The corrosion products are barely distinguishable with micro-CT (S2 Fig), but can be observed by comparing morphologies with micro-CT morphologies after removing corrosion product (Fig 2), especially at the position of pitting corrosion (eg. AZ31 with corrosion product and ZWEK-L with corrosion product). The elemental distribution of the degradation products on Mg-based alloy’s surface was analyzed using EDX mapping (S3 Fig). There are accumulated elements distributed around pitting corrosion which indicates corrosion products (Fig 5). At both static (Fig 5(a)) and dynamic (Fig 5(b)) conditions, dysprosium (Dy) deposition is observed on the surface of ZWEK-L and ZWEK-C because Dy_2_O_3_ is highly insoluble in water. The solubility product constant (Ksp) for Dy(OH)_3_ is 1.4 × 10^−22^, which is much smaller than that of Mg(OH)_2_ (5.61 × 10^−12^). Thus, Dy_2_O_3_ and Dy(OH)_3_ are more likely to remain in the corrosion layer than Mg(OH)_2_ due to the lower solubility [47].

Most of the elements of corrosion product layer consisted of Ca, P, Mg, and O, as previously reported [48]. The Mg-rich and O-rich layer was reported composed of MgO and/or Mg(OH)_2_ [49]. The Ca, P, O-rich zones on samples surface suggested that there was a formation of a calcium phosphate (Ca-P) layer on the Mg surface caused by the precipitation of the Ca^2+^ and HPO_4_^2-^ from DMEM [50]. The fast Mg degradation in dynamic condition, associated with pH shifts promotes the Ca-P precipitation [25]. Based on the Magnesium Pourbaix diagram (E_H_-pH diagram system of Mg-O-H), any Mg(OH)_2_ layer on a magnesium surface should have been dissolved in DMEM, where pH is lower than 10.5 [51]. The presence of Mg and O suggests the complex reactions on the Mg-based alloy’s surface. Possible reasons include: the local pH was higher than 10.5, or MgO and/or Mg(OH)_2_ was protected from the dissolution by Ca-P layer in the micro-environment, or they were dissolved and the Mg, O participated the formation of Mg-Ca apatite in the form of (Ca_1-x_Mg_x_)_10_(PO_4_)_6_(OH)_2_ [16, 52–55].

#### Element composition-secondary phase and impurities

**Fig 6:**
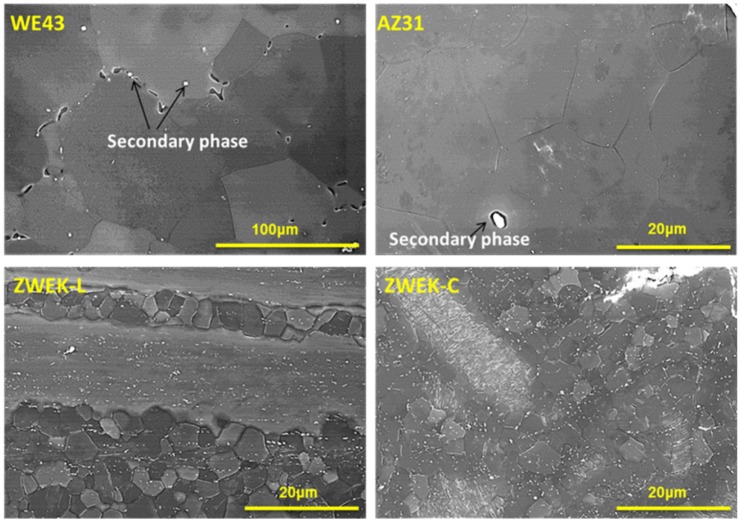
Grain structure of WE43, AZ31, ZWEK-L and ZWEK-C. Grain diameter (D) are: D(WE43) = 101±26μm, D(AZ31) = 15±7μm, D(ZWEK-L) = 5±1μm, D(ZWEK-C) = 5±2 μm.

**Fig 7:**
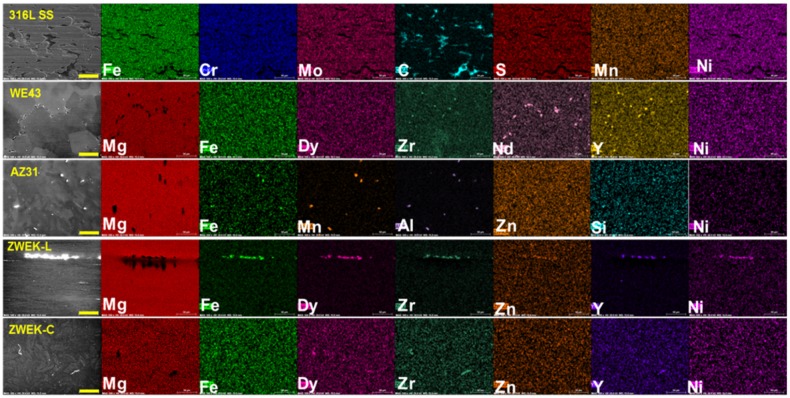
EDX mapping of aggregated elements. The aggregated elements were observed by EDX on the surface according to alloy’ fabrication procedure of 316L SS and Mg-based alloys. Scale bar = 50μm.

To determine the presence of secondary phase and impurities, grain structures of tested Mg-based alloys were observed using SEM (Fig 6) and elemental distribution using EDX mapping (Fig 7). The secondary phases are shown in SEM images as bright dots in WE43 and AZ31, and they are aligned as continuous network along grain boundaries in WE43. In WE43, there are microphores between grains due to casting defects. For ZWEK-L and ZWEK-C, the recrystallined and non-recrystallined structures make ZWEK alloy with two different textures [56, 57]. EDX mapping shows the elements accumulated in the secondary phases are: Zr, Nd and Y in WE43; Fe, Mn, Al in AZ31; Fe, Zr, Zn, Dy, Ca, Ni, Y in ZWEK-L; Dy and Zr in ZWEK-C. Among these elements, Fe and Ni are impurities.

The corrosion rate and corrosion morphologies (in section 3.1.1 Corrosion rate) revealed the low corrosion resistance of ZWEK-L and AZ31. The low corrosion resistance is due to the internal galvanic corrosion caused by the size of secondary phases or impurities exposed to fluid [58]. In Mg-based alloys, the secondary phases and impurities have more noble potential than the Mg matrix and act as local micro-cathodes. The Mg matrix acts as the anode. Local micro-galvanic currents occur causing increased corrosion rate [59]. When the secondary phases are in the form of continuous network along grain boundaries, they are able to act as barriers against corrosion [60]. The continuous network is one reason for higher corrosion resistance of WE43. For both ZWEK and WE43, Zr is added to refine grain and Zr-rich regions in the center of the grains enhance corrosion resistance for these regions as compared with the remainder of the grain [61–63]. Fe impurities dominate the corrosion of Mg-based alloys, if their concentration is above a tolerance limit (about 180 ppm) [64]. In this case, body-centered cubic Mg–Fe precipitates are sites of cathodic hydrogen evolution [65]. Impurities, such as Cu, Ni, and Fe affect detrimentally the corrosion behavior of Mg, while others such as Al, Zn or Mn hardly influence the corrosion resistance of the alloys or even decrease the corrosion rate of Mg itself [13, 66–68]. In EDX images, both AZ31 and ZWEK-L have Fe, so they have lower corrosion resistance than WE43 and ZWEK-C, where there are no such impurity elements.

Impurities and secondary phases are elongated along extrusion direction [69–73]. Larger areas of impurities and secondary phases of ZWEK-L are exposed to medium than ZWEK-C (Fig 7), act as larger cathode surface of ZWEK-L than that of ZWEK-C. According to the surface area effect (i_a_ = i_c_ S_c_/S_a_), the larger surface area ratio is a serious aggravating factor for corrosion [74]. Surface effect is one reason for ZWEK-L corroded faster than ZWEK-C though they have the same chemical composition. Also, it was observed the pits were observed on LAE442 rod surface to be more frequently aligned to the extrusion direction (32). However, we did not observe this phenomenon due to the presence of impurities.

### Thrombosis assessment

#### Platelets adhesion

To evaluate the initial stage of thrombosis potential of Mg-based alloy, platelets adhesion test was conducted in the microfluidic system. The blood as a dynamic flow not only exerts shear stress to the device, its components affect the corrosion behavior of the Mg-based implants. Blood cells adhesion to stents is the first stage that leads to thrombosis and slows down corrosion rate [75]. The fewer platelets adhesion on alloys surface is the initial indication for less thrombogenic potential.

**Fig 8:**
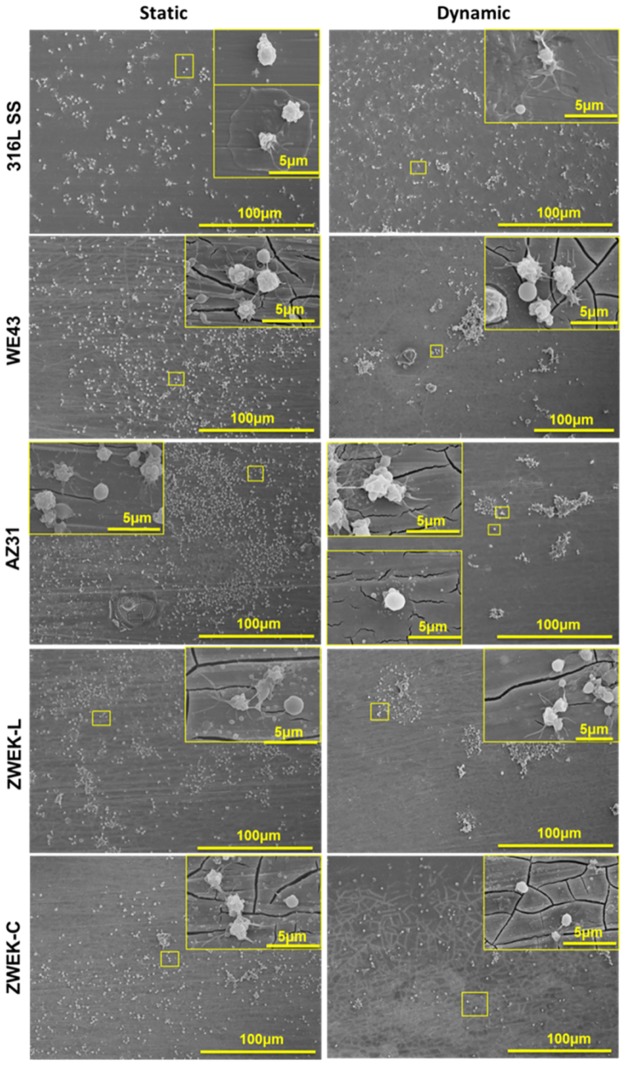
Platelets adhesion morphology. The platelets adhesion morphologies were observed at the surface of stainless steel (316L SS) and four Mg-based alloys (WE43, AZ31, ZWEK-L, and ZWEK-C) in both static and dynamic platelet rich plasma.

**Fig 9:**
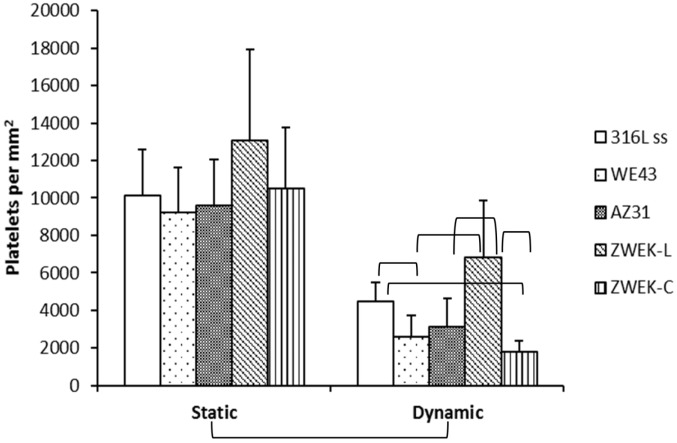
Platelets adhesion rate at both static and dynamic condition. Parenthesis mean significant differences between groups (P<0.05).

Platelets adhesion on the tested samples was observed by SEM (Fig 8). The platelet adhesion on alloys surface in static condition is significantly more than that in dynamic condition (Fig 9, P<0.05). Aggregated activation is obviously observed in dynamic condition(Fig 8). The lower adhesion rate on Mg-based alloys surface in dynamic condition is due to the shear stress. The shear stress induced a dragging force to pull platelets away from alloys surface.

Comparing platelets adhesion rate of different alloys (Figs 8 and 9), in static condition, there is no significant difference between alloys (P>0.05), but in dynamic condition, there are significant differences between some alloys are observed: 316L SS vs WE43, 316L SS vs ZWEK-C, ZWEK-L vs WE43, ZWEK-L vs AZ31, ZWEK-L vs ZWEK-C. The platelets adhesion rate of Mg-based alloys in dynamic condition has a similar pattern with corrosion rate (Fig 3): ZWEK-L> AZ31 > WE43 ≈ZWEK-C.

#### Particulate analysis

To analyze the potential of particulates generation of biodegradable alloys, the sediment possibly containing particulate was collected by centrifuge of circulated DMEM. The element distributions of all tested samples are shown in S4 Fig. Mg, O, Ca, P were in the particulate of WE43, AZ32, ZWEK-L and ZWEK-C more obviously compared with that in 316L SS. Zn is in all samples because it comes from the culture medium. This element composition in the particulate is similar with that in corrosion product at corroded Mg-based alloys surface (Fig 5). So a similar behavior for decomposition processes of Mg corrosion product, most possibly, (Ca_**1 −***x*_Mg_*x*_)_10_(PO_4_)_6_OH_2_, can be expected [76].

The microfluidic system allows analysis of particulate generated by Mg-based alloys degradation, which is rarely studied in current bench experiment. Biodegradable Mg-based alloys are designed to degrade gradually *in vivo*, with an appropriate host response elicited by released corrosion products, then dissolve completely upon fulfilling the mission to assist with tissue healing, ideally with no implant residues [77]. The residues exist because of the poor hemocompatibility and uncontrolled degradation behaviors. Before the Mg-based alloy totally corrodes, the dislodged particles and corrosion product could be washed downstream and circulate with blood flow in the vessel, and recruit platelets, resulting in downstream emboli.

### Degradation and thrombosis

**Fig 10:**
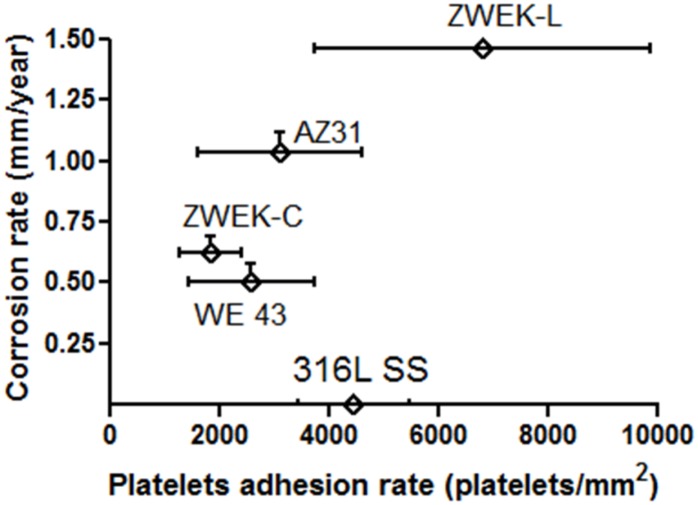
The relation between corrosion rate and platelets adhesion. The graph was plotted in Prism 5.

After we got the outcomes of corrosion rate (Fig 3) and platelets adhesion rate (Fig 9), we found they have the same trend at dynamic condition within Mg-based alloys. Corrosion rate of 3 days exposure in DMEM and platelets adhesion rate of 3 hours exposure in PRP were plotted in same figure (Fig 10). This results indicating the higher degradability associated with higher thrombogenic potential of Mg-based alloys. Since 316L SS does not corrode and does not change pH, [Mg2+] or hydrogen, it was plotted in the graph as a control. This phenomenon is believed to be related to pH, [Mg^2+^] and hydrogen release associated with equation Mg+2H_2_O→Mg(OH)_2_+H_2_ and Mg(OH)_2_⇔ Mg^2+^+2OH^-^.

Firstly, Chaimoff et al showed that change of the pH from acidosis to 7.0 and even to slight alkalosis induces platelets aggregation, platelet calcium, serotonin release, and platelet factor III availability [78]. The corrosion of Mg-based alloys increased the localized pH, which was higher corrosion rate higher localized pH. The localized pH cannot be represented by the medium pH. The pH of the complete medium after corrosion test increased (S5 Fig), and no significant difference between alloys in dynamic condition. However, we did not observe pH increase at platelets adhesion experiment (pH<7.6) since we only incubated 3 hours, during which time corrosion was not affected.

Secondly, higher corrosion rate, higher Mg ion released. Mg^2+^ promotes cell adhesion depending on 5β1- and β1-integrins associated signal transduction pathways, involving enhanced activation of the key signaling adaptor protein Shc (Src homology collagen) and results in enhanced gene expression of extracellular matrix proteins [79, 80]. Mg ion has been reported in animal trials, producing a significant reduction in acute stent thrombus formation that was equivalent in magnitude to that produced by tirofiban and eptifibatide [81].

Last but not least, hydrogen release was deduced to promote platelets adhesion, because the increased gas nuclei may affect the proteins and direct contact with platelet [29, 82]. There is the more rapid release of gas bubbles with higher corrosion rate in dynamic condition than in static condition. H_2_ was taken away by hemodynamics. We only observed bubbles in static incubation. Thus H_2_ is not a dominant factor affecting platelet adhesion in dynamic condition. In static condition, H_2_ release plays an important role in platelet adhesion because some micro-gas bubbles will stay on the surface of Mg-based alloys to prevent platelet adhesion. The alloys with higher corrosion rate have more micro-gas bubbles on the surface, leading to less free occupation for platelets attachment. This probably is the reason the alloys with higher corrosion rate, but fewer platelets adhesion in some static studies [28, 29].

Surface roughness is another reason to explain the relation between corrosion behavior and platelets attachment. It has been studied that platelet adhesion was not affected by surface roughness in static condition, but in a laminar flow, it increased with the addition of roughness on the hydrophobic surface [83]. The surface of the Mg-based alloys is rougher with higher corrosion rate, so more platelets attached on the surface expressing higher potential to get thrombosis.

## Conclusion

The microfluidic-based *in vitro* system allows the assessment of biodegradability and thrombosis potential of Mg-based alloys in dynamic DMEM and platelet rich plasma, respectively. The flow-induced shear stress is the indispensable condition to evaluate Mg-based alloys for stent application because of the significance of dynamic effect on corrosion and platelets adhesion. Among the evaluated Mg-based alloys, WE43 has the highest corrosion resistance and least thrombogenic potential. Biodegradability characterized by corrosion rate, type and products and dominated by elemental composition. Particularly, the presence of impurities and secondary phases introduce galvanic corrosion to alloys and lower the corrosion resistance. Thrombosis potential initially indicated by platelet attachment also varies between different alloys. The corrosion rate is found the same trend with the platelets adhesion rate of the four tested Mg-based alloys, indicating higher biodegradability, higher initial thrombosis potential. The novelty of this research is the microfluidics-based test bed, which allows dynamic test of corrosion behavior, especially allows dynamic test of platelets adhesion.

The future study includes the design and fabrication of high-throughput microfluidic system which will allow screening the quality of stents materials in a physically-simulated *in vitro* microenvironment. The limitation of this study is tissue responses to the particulate were not able to be analyzed and there is a high possibility that the circulated particulates will be encapsulated by endothelial cells. Future studies will also include endothelialization with cardiovascular-related cell lines.

## Supporting information

S1 Fig Geometry and computational fluid dynamics (CFD) contours. (a) Geometry meshes of a microfluidic chip. (b) Surface velocity (m/s) magnitude contour in DMEM complete medium. (c) Surface velocity (m/s) magnitude contour in PRP. The mathematical model assumed an incompressible and isotropic Newtonian fluid. The flow chamber is with length (l) of 50 mm, width (b) of 5mm and height (h) of 400 μm. More than 85% of the surface is exposed to a homogenous wall shear stress because of b/h>20 (1). The inlet boundary condition has a laminar flow with a 0.15m entrance length (calculated with Lentry = 0.04h Re, Reynolds number Re = Qρ/(μb)), and outlet boundary condition has a laminar outflow with zero pressure and a 0.15m exit length. For the mesh (Fig S1 (a)), automatical mesh and tetrahedral elements were used for channel region and inlet and outlet tubes, as well as the three alloy blocks. The domains of fluid chamber consist of 4473 elements and the domains of three alloys blocks consist of 93 elements. Overall, the mesh consists of 4566 elements. Computer fluid dynamics (Fig S1 (b) and (c)) showed that the samples region was exposed to laminar flow.
(TIF)

S2 Fig Micro-CT images. Micro-CT image of surface morphology (Top, Front, Side and 3D view) of degraded alloys at both static and dynamic conditions before removing corrosion product. The corrosion products are observed as light gray area. Scale bar = 1.5mm.
(TIF)

S3 Fig EDX mapping for full sized alloys. EDX images of elements distribution at the surface of 316L ss and Mg-based alloys after 3 days at static and dynamic conditions. Scale bar = 1mm.
(TIF)

S4 Fig EDX mapping for particulate analysis. Common elemental (Mg, O, Ca, P and Zn) distribution on the particulate of tested alloys is shown with EDX mapping.
(TIF)

S5 Fig PH of alloys in dynamic condition and total pH in static condition. The alloys were immersed in one container in static condition, so the pH reflects the total pH of all alloys together.
(TIF)
