# Supplementary figures and images for: Biodegradability and platelets adhesion assessment of magnesium-based alloys using a microfluidic system
Source: PLoS One. 2017 Aug 10;12(8):e0182914. doi: 10.1371/journal.pone.0182914 (PMC5552284; doi:10.1371/journal.pone.0182914)

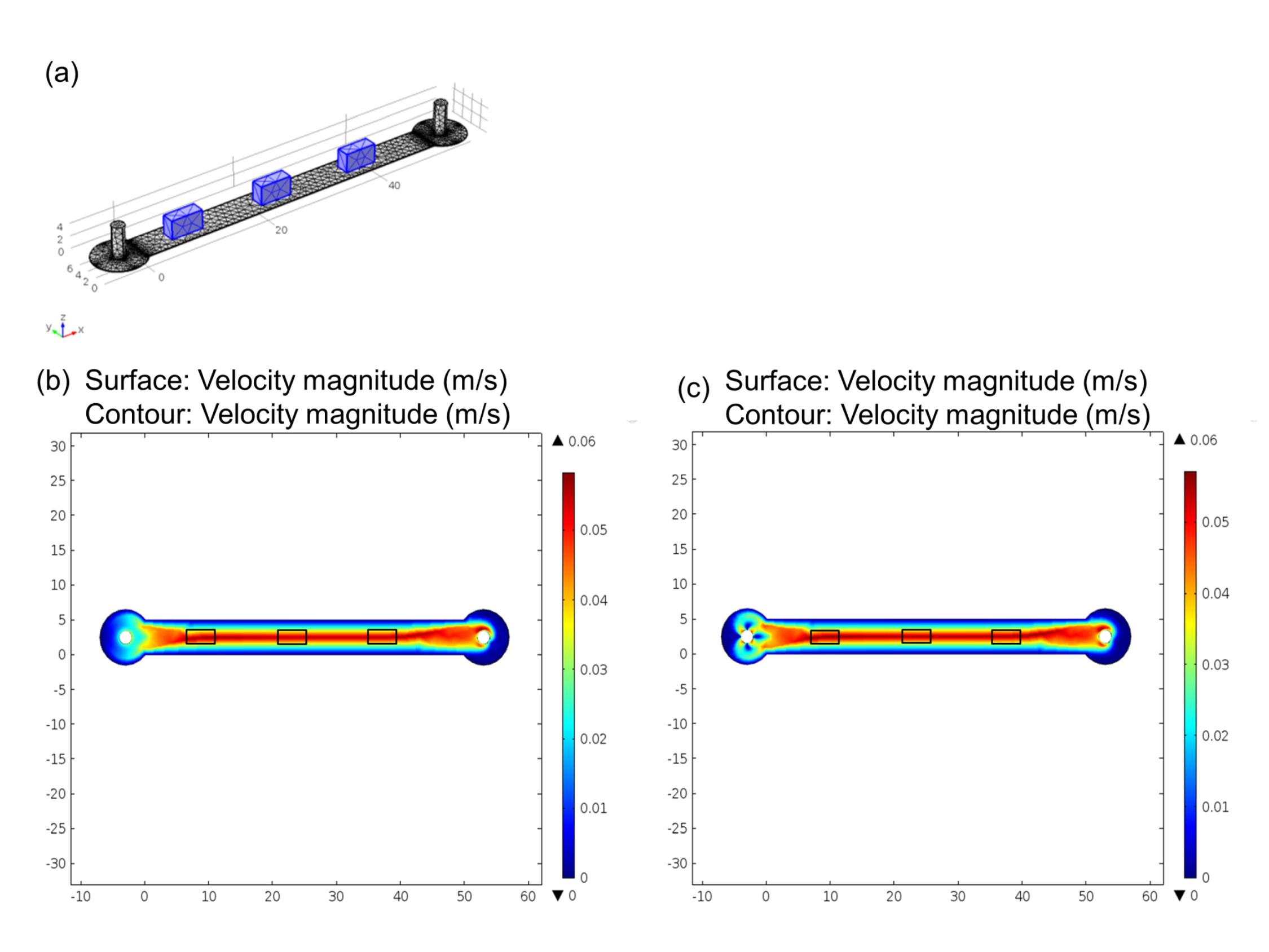

Supplement: S1 Fig — (a) Geometry meshes of a microfluidic chip. (b) Surface velocity (m/s) magnitude contour in DMEM complete medium. (c) Surface velocity (m/s) magnitude contour in PRP. The mathematical model assumed an incompressible and isotropic Newtonian fluid. The flow chamber is with length (l) of 50 mm, width (b) of 5mm and height (h) of 400 μm. More than 85% of the surface is exposed to a homogenous wall shear stress because of b/h>20 (1). The inlet boundary condition has a laminar flow with a 0.15m entrance length (calculated with Lentry = 0.04h Re, Reynolds number Re = Qρ/(μb)), and outlet boundary condition has a laminar outflow with zero pressure and a 0.15m exit length. For the mesh (Fig S1 (a)), automatical mesh and tetrahedral elements were used for channel region and inlet and outlet tubes, as well as the three alloy blocks. The domains of fluid chamber consist of 4473 elements and the domains of three alloys blocks consist of 93 elements. Overall, the mesh consists of 4566 elements. Computer fluid dynamics (Fig S1 (b) and (c)) showed that the samples region was exposed to laminar flow. (TIF) [file pone.0182914.s001.tif]

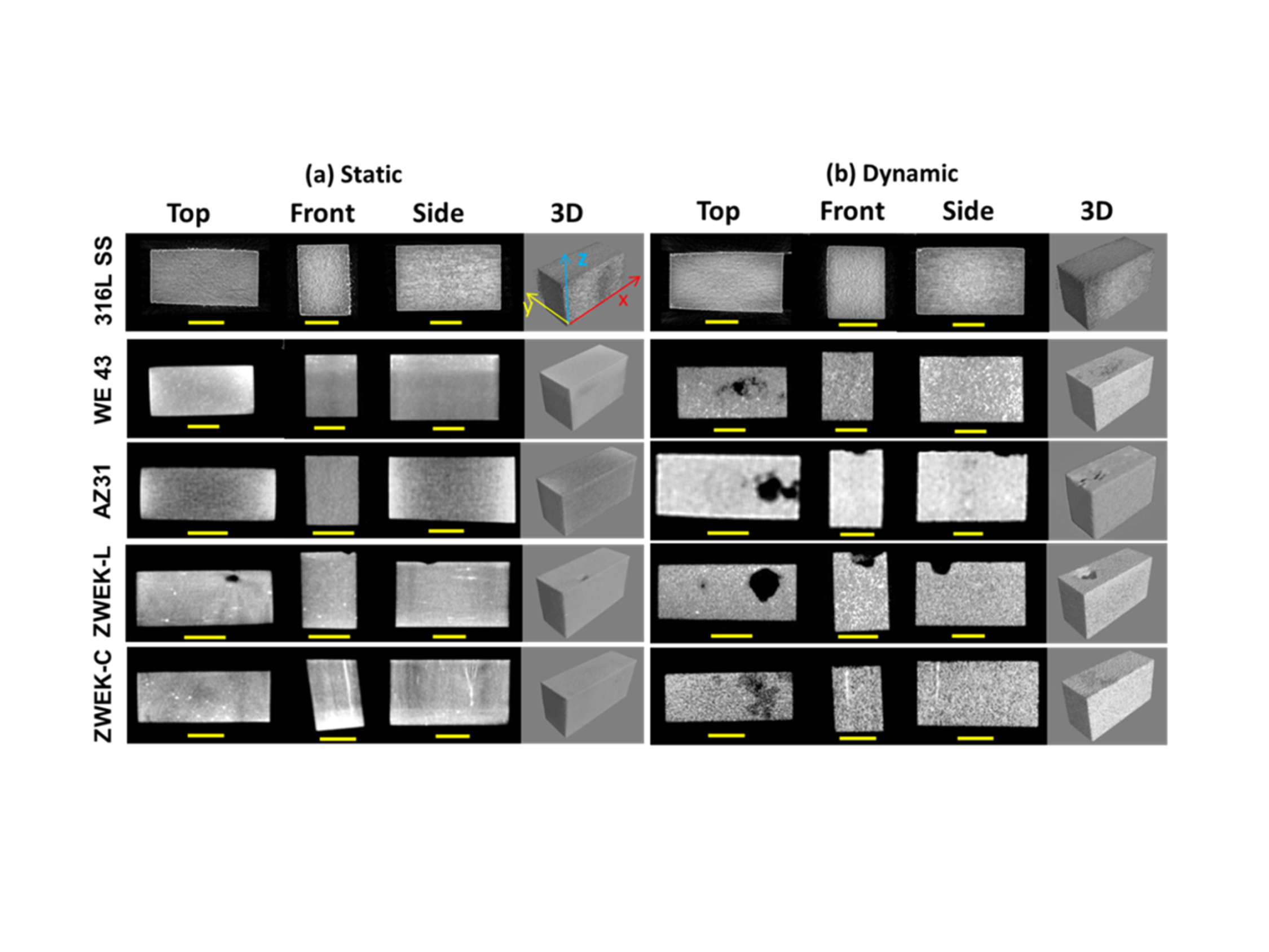

Supplement: S2 Fig — Micro-CT image of surface morphology (Top, Front, Side and 3D view) of degraded alloys at both static and dynamic conditions before removing corrosion product. The corrosion products are observed as light gray area. Scale bar = 1.5mm. (TIF) [file pone.0182914.s002.tif]

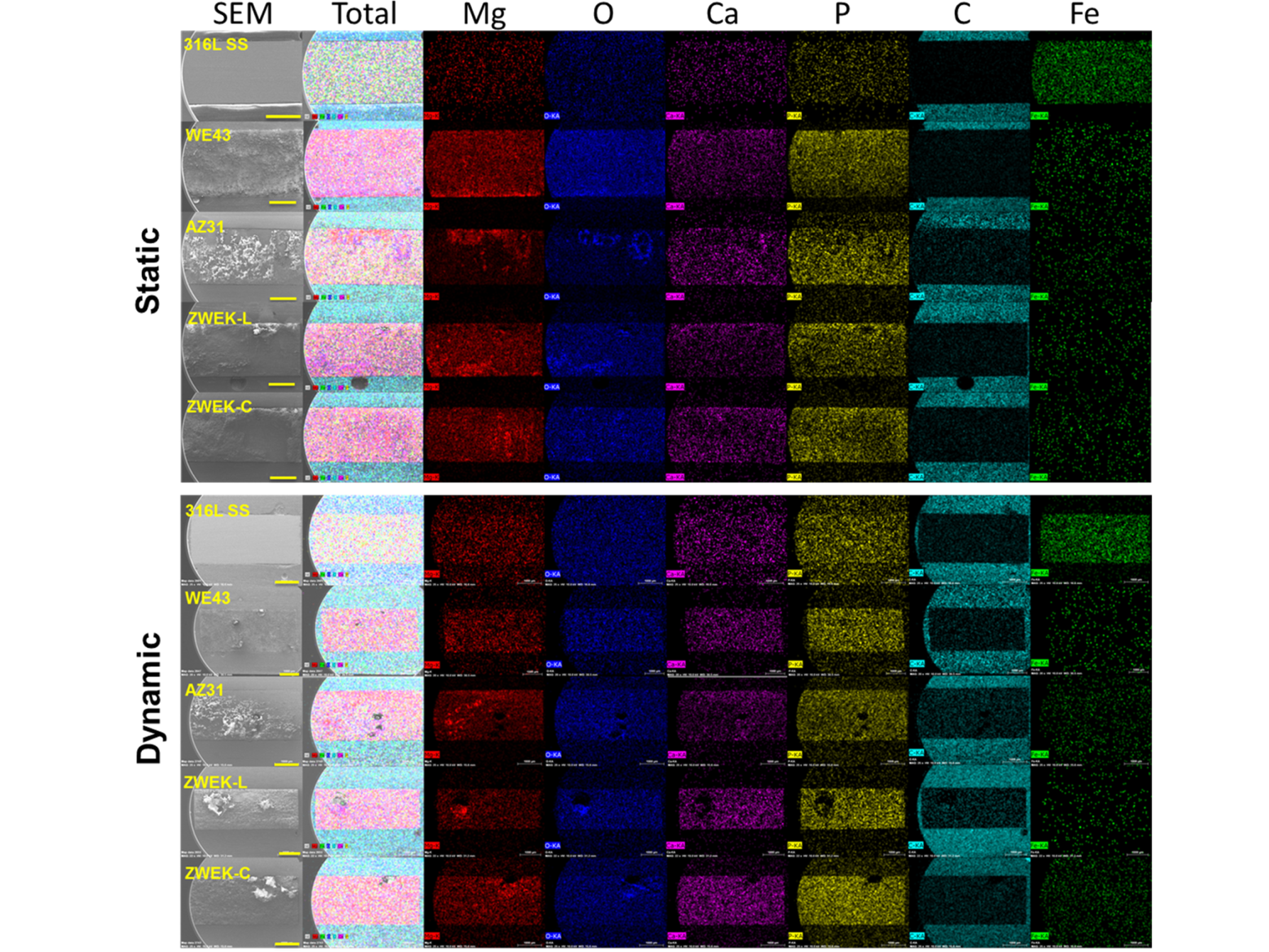

Supplement: S3 Fig — EDX images of elements distribution at the surface of 316L ss and Mg-based alloys after 3 days at static and dynamic conditions. Scale bar = 1mm. (TIF) [file pone.0182914.s003.tif]

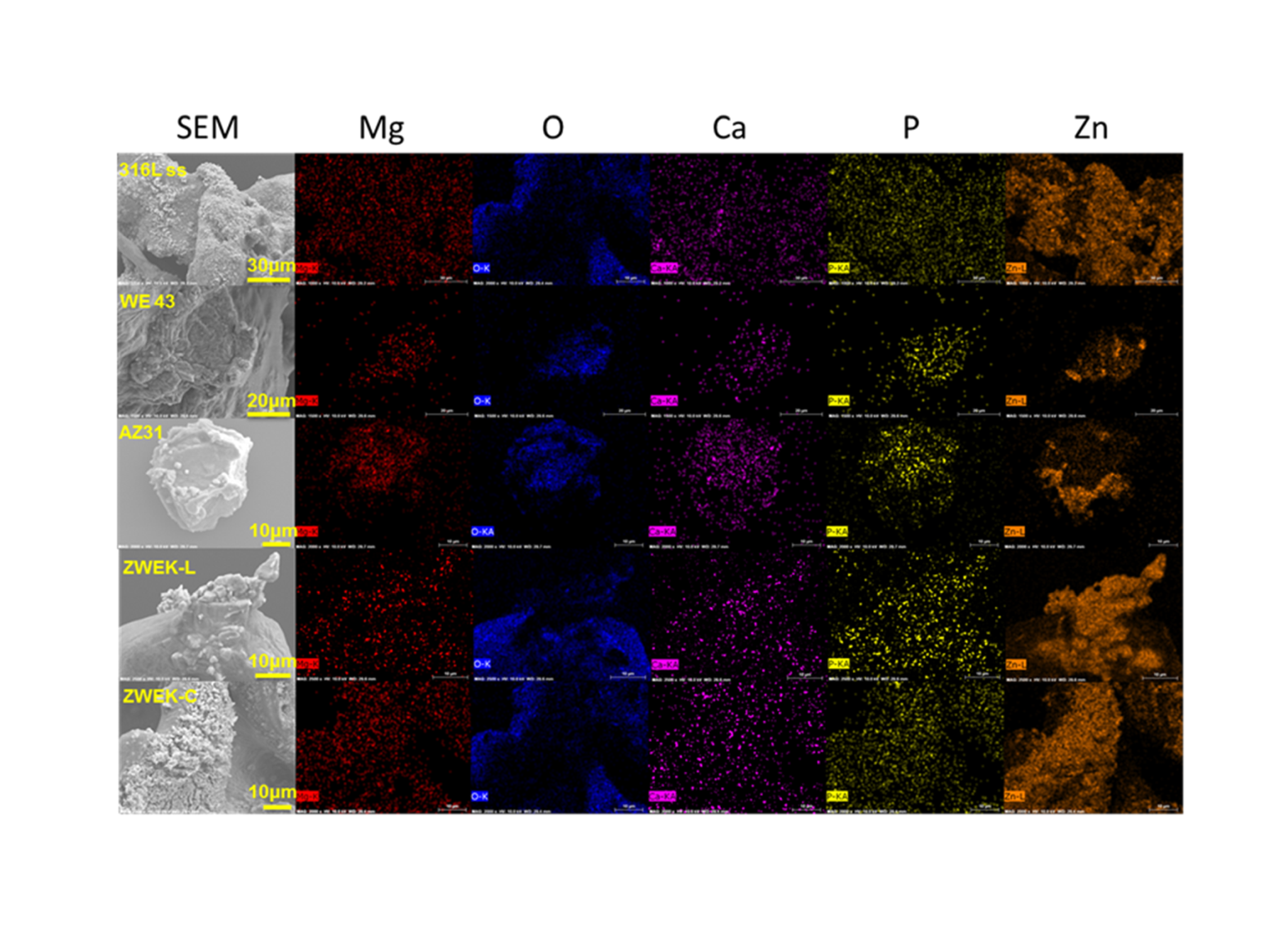

Supplement: S4 Fig — Common elemental (Mg, O, Ca, P and Zn) distribution on the particulate of tested alloys is shown with EDX mapping. (TIF) [file pone.0182914.s004.tif]

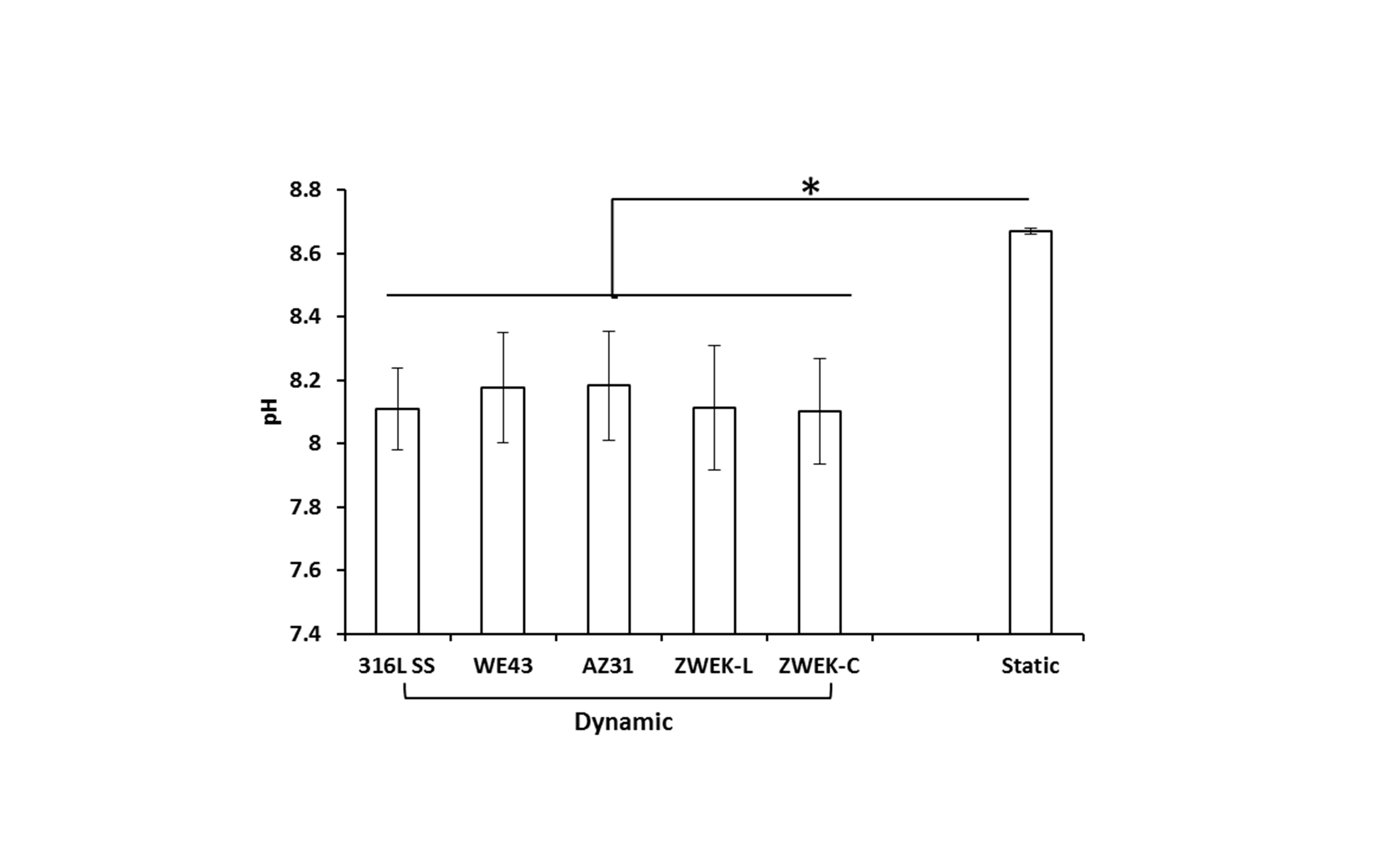

Supplement: S5 Fig — The alloys were immersed in one container in static condition, so the pH reflects the total pH of all alloys together. (TIF) [file pone.0182914.s005.tif]
